# Recruiting marijuana-exposed maternal-infant dyads for longitudinal study: a feasibility assessment

**DOI:** 10.3389/fpsyt.2025.1637076

**Published:** 2025-09-12

**Authors:** Nayana A. Sojin, Rhea Parimoo, Lauren A. Agliano, Amie J. Goodin, Deepthi S. Varma, Bruce A. Goldberger, Ibrahim Tuna, Reem Abu-Rustum, Kay Roussos-Ross

**Affiliations:** ^1^ Department of Obstetrics and Gynecology, University of Florida College of Medicine, Gainesville, FL, United States; ^2^ Pharmaceutical Outcomes and Policy, Center for Drug Evaluation and Safety, Consortium for Medical Marijuana Clinical Outcomes Research, University of Florida College of Pharmacy, Gainesville, FL, United States; ^3^ Department of Epidemiology, University of Florida College of Public Health & Health Professions and College of Medicine, Gainesville, FL, United States; ^4^ Department of Pathology, Immunology and Laboratory Medicine, University of Florida College of Medicine, Gainesville, FL, United States; ^5^ Department of Neuroradiology and Pediatric Radiology, University of Florida College of Medicine, Gainesville, FL, United States

**Keywords:** marijuana, pregnancy, perinatal substance use, neonates, perinatal care

## Abstract

**Introduction:**

*In-utero* marijuana exposures are correlated with adverse neurodevelopmental outcomes in exposed neonates. As rates of marijuana use in pregnancy and postpartum reportedly increase, it is crucial to obtain conclusive, pregnancy-specific safety data through well-designed clinical research studies. The objective of this study is to assess feasibility of recruitment and retention of marijuana-exposed pregnant women for longitudinal study involving biospecimen and imaging collection.

**Methods:**

Participants self-reporting marijuana use in pregnancy and controls with no self-reported exposure were recruited from routine prenatal care in a large health-system. Consented participants completed imaging and biological specimen collections during pregnancy, at delivery, and postpartum. Proportions of collected samples/images at each data collection interval were calculated and compared for exposed versus unexposed.

**Results:**

30 participants were recruited over 20 months: 77% (n=23) self-reported as marijuana-exposed and 23% (n=7) reported as unexposed (control). 70% (n=21) of participants completed the study (n=14 marijuana-exposed; n=7 control), while 30% (n=9 marijuana-exposed; 0%, n=0 control) completed some study visits before becoming lost-to-follow-up (LTFU).

**Discussion:**

Preliminary findings suggest that it is feasible to recruit and retain pregnant women using marijuana for longitudinal study. Although marijuana-exposed participants were more likely than control participants to miss postpartum visits, become LTFU, and require rescheduling of study visits, marijuana-exposed participants were still found to complete 68% of study visits.

## Introduction

Marijuana is the most frequently used illicit substance among pregnant women (1). According to the National Survey on Drug Use and Health (NSDUH), the prevalence of pregnant women who self-reported last month marijuana use increased from 3.4% to 7.1% between 2015 and 2018 (1).

Although current evidence establishing causal links between *in-utero* cannabinoid exposure and adverse outcomes in exposed neonates remains limited, the American College of Obstetricians and Gynecologists (ACOG) and the American Academy of Pediatrics (AAP) both recommend avoidance of marijuana products during years of reproductive potential, pregnancy, and breastfeeding (2, 3). Emerging evidence suggests several possible adverse outcomes from perinatal cannabis use on exposed offspring, including but not limited to: adverse birth outcomes such as low birth weight or premature birth (4–6); behavioral effects such as attention/impulse control deficits (7); communication delays (8); learning delays (9); and structural brain changes associated with impaired executive functioning and neural connectivity (10). Preclinical studies in animal models mirror these neurodevelopmental findings in human research, suggesting further research is needed to fully elucidate potential effects on cognition (e.g., memory, attention, and spatial learning deficits), structural brain changes (discrepancies in brain volume, blood flow, and ventricular size), and neuroplasticity disruption (11–14).

Multiple states and jurisdictions in the United States have legalized medicinal and/or recreational marijuana use over the last decade, with a handful of states also decriminalizing marijuana possession and use. This study was conducted in the state of Florida, where medical marijuana use was legalized in 2016 per the Medical Marijuana Act, though recreational marijuana use still remains prohibited (15). As more cannabinoid products continue to become available, one notable consumer group is that of pregnant/postpartum individuals, who may also consider health effects on a developing fetus when weighing risks and benefits of cannabinoid use. Since risks of resulting adverse neonatal outcomes are not yet fully quantified as a function of marijuana and other cannabinoid (e.g., CBD) exposures, and perinatal marijuana use rates continue to increase, there is a growing need for the longitudinal assessment of maternal and fetal outcomes following exposures during pregnancy. Longitudinal assessment requires well-designed clinical research studies that follow maternal-infant dyads over the perinatal period. This study evaluates the feasibility of recruiting and retaining marijuana-exposed maternal-infant dyads for perinatal imaging and biospecimen collection.

## Methods

### Study population and recruitment

30 participants were enrolled between July 2023 and February 2025. Participants were recruited from inpatient and outpatient OBGYN settings at an academic health system in Florida. Recruitment was conducted following screening for self-reported marijuana use during obstetric visits. Eligibility criteria included pregnant patients aged 18–50 who spoke English and self-reported perinatal marijuana exposure. Patients with no marijuana exposure were recruited as controls. Obstetric care clinicians assessed patient interest and subsequently referred patients to the study coordinator for enrollment.

Participants were enrolled in any trimester. All participants were consented using an IRB-approved protocol (IRB#202300712) to protect patient confidentiality and informed consent was obtained. Demographic information was collected at enrollment: patient age, parity, gestational age at enrollment, race, educational level, insurance status, history/current use of marijuana or CBD, mode/frequency of marijuana/CBD use, and if the participant owned a medical marijuana card.

### Data collection and analysis

Biospecimen collection included maternal urine in each trimester; placenta, umbilical cord, maternal urine, and neonatal meconium at delivery; and postpartum maternal urine and breastmilk. Biospecimens were analyzed for natural and synthetic cannabinoids and metabolites utilizing liquid chromatography-tandem mass spectrometry (16–18). Imaging included fetal ultrasound each trimester and a third trimester fetal magnetic resonance imaging (MRI). Imaging assessed fetal growth, head circumference, cerebral blood flow, and placental volume. Feasibility assessment included calculating retention proportions via completed collections (e.g., losses-to-follow-up per biospecimen type). Barriers and facilitators to participation were determined via participant self-reports regarding study visit non-attendance, and emerging themes for missed visits were identified. Additionally, trends in biospecimen/imaging collection, visit completion rates, and losses-to-follow-up were analyzed to isolate factors influencing study participation.

## Results

**Figure 1:**
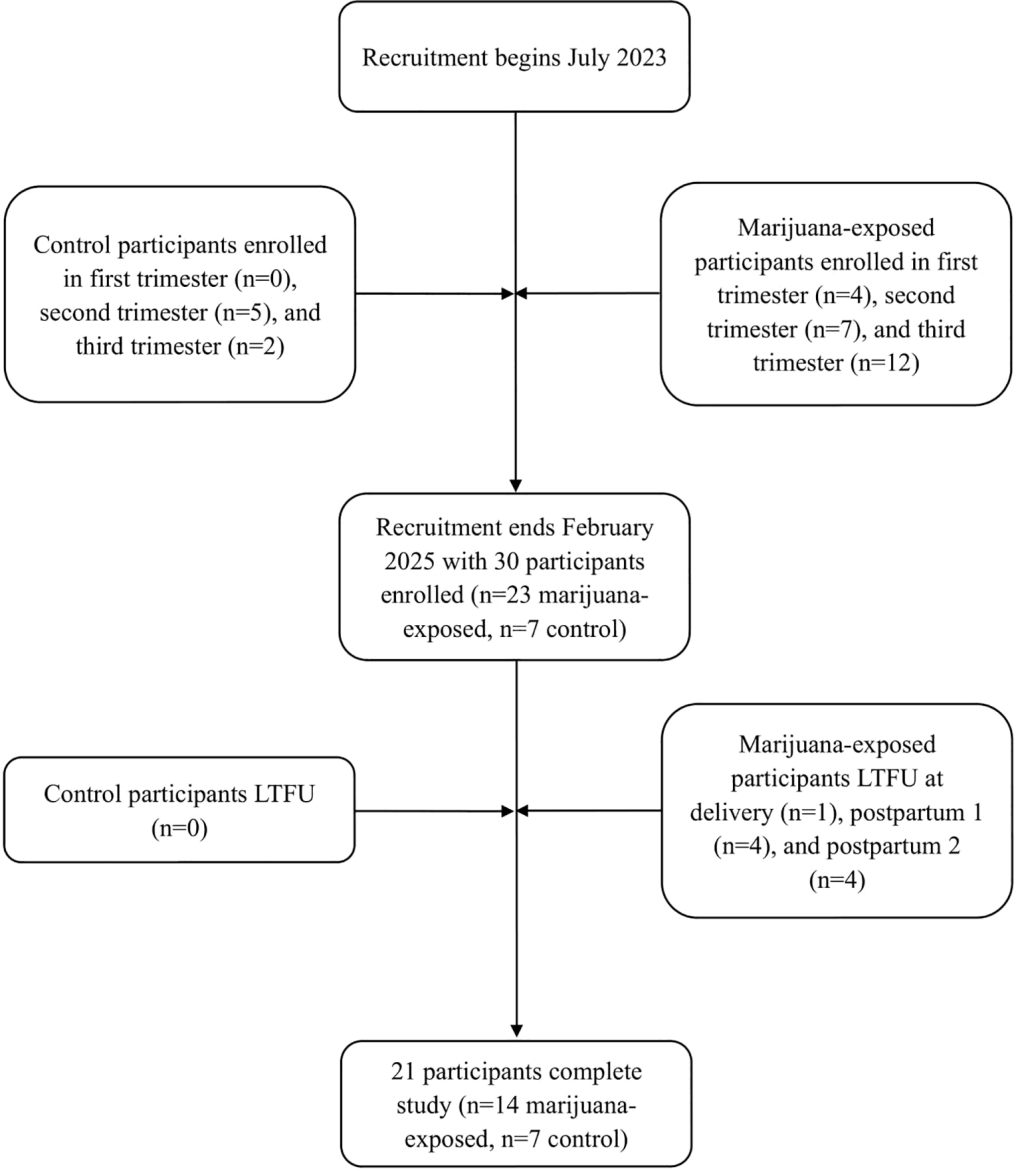
Flowchart of participant enrollment and retention. LTFU, lost-to-follow-up; postpartum 1, 1–4 weeks postpartum; postpartum 2, 4–8 weeks postpartum.

Out of 30 total participants, 77% (n=23) self-reported as marijuana-exposed and 23% (n=7) reported as unexposed (**Figure 1**). Of the 23 participants self-reporting as marijuana-exposed, 91% (n=21) identified as Caucasian, 4% (n=1) identified as Hispanic, and 4% (n=1) identified as Black. 35% (n=8) of exposed participants had less than a high school education, 39% (n=9) graduated high school, and 26% (n=6) received higher education. 30% (n=7) of marijuana-exposed participants reported owning a medical marijuana card. Additionally, 74% (n=17) had Medicaid as primary insurance and 26% (n=6) reported “private insurance” or “other”. Proportions of biospecimen/imaging completion are shown in **Tables 1** and **2**.

**Table 1:** Biospecimen samples completed by exposed and control participants.

Collection window^§^	Biospecimen type									
	Urine		Placenta		Umbilical cord		Meconium		Breastmilk	
	Exposed	Control	Exposed	Control	Exposed	Control	Exposed	Control	Exposed	Control
Trimester 1	66.7% (2 out of 3)	0.0% (0 out of 0)	–	–	–	–	–	–	–	–
Trimester 2	90.9% (10 out of 11)	60.0% (3 out of 5)	–	–	–	–	–	–	–	–
Trimester 3	90.9% (20 out of 22^±^)	100.0% (7 out of 7)	–	–	–	–	–	–	–	–
Delivery	69.6% (16 out of 23)	100.0% (7 out of 7)	73.9% (17 out of 23)	100.0% (7 out of 7)	73.9% (17 out of 23)	85.7% (6 out of 7)	60.9% (14 out of 23)	71.4% (5 out of 7)	–	–
Postpartum 1	78.2% (18 out of 23)	85.7% (6 out of 7)	–	–	–	–	–	–	70.6% (12 out of 17^*^)	100.0% (5 out of 5^*^)
Postpartum 2	56.5% (13 out of 23)	100.0% (7 out of 7)	–	–	–	–	–	–	41.2% (7 out of 17^*^)	100.0% (5 out of 5^*^)
Total	75.2% (79 out of 105)	90.9% (30 out of 33)	73.9% (17 out of 23)	100.0% (7 out of 7)	73.9% (17 out of 23)	85.7% (6 out of 7)	60.9% (14 out of 23)	71.4% (5 out of 7)	55.9% (19 out of 34)	100.0% (10 out of 10)

^§^collection windows include the following: trimester 1 (1–12 weeks), trimester 2 (13–27 weeks), trimester 3 (28–40 weeks), delivery (during the delivery hospitalization), postpartum 1 (1–4 weeks postpartum), postpartum 2 (4–8 weeks postpartum).

^±^n=1 marijuana-exposed participant was enrolled in the third trimester (39 weeks) during delivery hospitalization, and was not eligible for any antenatal biospecimens/imaging.

^*^n=6 marijuana-exposed and n=2 control participants did not breastfeed postpartum.

**Table 2:** Imaging completed by exposed and control participants.

Collection window^§^	Imaging type			
	Ultrasound		Fetal MRI^±^	
	Exposed	Control	Exposed	Control
Trimester 1	66.7% (2 out of 3)	0.0% (0 out of 0)	–	–
Trimester 2	87.5% (7 out of 8)	60.0% (3 out of 5)	–	–
Trimester 3	85.0% (17 out of 20^*†^)	100.0% (7 out of 7)	68.2% (15 out of 22^*^)	100.0% (7 out of 7)
Total	83.9% (26 out of 31)	83.3% (10 out of 12)	68.2% (15 out of 22)	100.0% (7 out of 7)

^§^collection windows include the following: trimester 1 (1–12 weeks), trimester 2 (13–27 weeks), and trimester 3 (28–40 weeks).

^±^ MRI, magnetic resonance imaging.

^*^n=1 marijuana-exposed participant was enrolled in the third trimester (39 weeks) during delivery hospitalization, and was not eligible for any antenatal biospecimens/imaging.

^†^n=2 marijuana-exposed participants were enrolled after 36 weeks, and were not eligible for ultrasound as accurate measurements were not able to be obtained.

### Biospecimens

In the antenatal period, at least one urine sample was collected from 87% (n=20) of marijuana-exposed participants. 4 participants were enrolled in the first trimester, 7 participants in the second trimester, and 12 participants in the third trimester; thus, out of 36 total possible urine sample collections, 89% (n=32) were collected.

At delivery, 78% (n=18) of marijuana-exposed participants completed at least one biospecimen. Maternal urine was collected from 70% (n=16) of participants, placenta from 74% (n=17), umbilical cord from 74% (n=17), and neonatal meconium from 61% (n=14). Biospecimens for 17% (n=4) of participants were uncollected due to missed identification of study participation on the labor and delivery unit by hospital staff. Similarly, 35% (n=8) of meconium samples were uncollected on the mother and baby unit. 4% (n=1) of marijuana-exposed participants were lost-to-follow-up (LTFU) at delivery.

In the postpartum period, at least one biospecimen was collected from 78% (n=18) of marijuana-exposed participants. 78% (n=18) provided a 1–3 weeks postpartum urine sample and 57% (n=13) provided a 4–6 weeks postpartum urine sample. 26% (n=6) of participants self-reported not breastfeeding; 71% (n=12) of breastfeeding participants provided a 1–3 weeks postpartum breastmilk sample and 41% (n=7) provided a 4–6 weeks postpartum breastmilk sample. 35% (n=8) of participants were LTFU in postpartum.

### Imaging

In the antenatal period, at least one fetal ultrasound was performed for 74% (n=17) of marijuana-exposed participants. The number of possible fetal ultrasounds was dependent on participant gestational age at enrollment, with one ultrasound performed per trimester. Out of 31 total possible fetal ultrasound collections, 84% (n=26) were completed. 68% (n=15) of marijuana-exposed participants completed a third trimester fetal MRI.

### Controls

Of the 7 control participants, 5 were enrolled in the second trimester and 2 in the third trimester. 60% (n=3) and 50% (n=2) of controls completed a second trimester urine sample and ultrasound, respectively. 100% (n=7) completed a third trimester urine sample, ultrasound, and MRI. At delivery and postpartum, at least one biospecimen was collected from 100% (n=7) of controls. 29% (n=2) self-reported not breastfeeding; 100% (n=5) of breastfeeding controls completed all postpartum samples.

### Barriers and facilitators

Recruitment and retention facilitators included financial compensation, rapport-building between participant and study coordinator, scheduling study visits onto existing obstetrics appointments, and promptly contacting patients to reschedule cancellations. Barriers included missed identification of study participation and biospecimen collection on the hospital unit due to staff being unfamiliar with study protocols. Other major obstacles, as described by participants, included access to transportation, finances, time constraints, and childcare.

## Discussion

Findings suggest that it is feasible to recruit and retain pregnant women using marijuana for longitudinal study. Although marijuana-exposed participants completed 68% of study visits, 43% (n=10) either canceled or required rescheduling of at least one appointment, compared to 0% (n=0) of controls. 39% (n=9) of marijuana-exposed participants were LTFU, with the majority (89%, n=8) occurring postpartum. Notably, no LTFUs were observed in the control group. Additionally, 39% (n=9) of exposed participants canceled, did not attend, or did not schedule their postpartum appointment, compared to 0% (n=0) of controls. This trend parallels current ACOG statistics which illustrate that up to 40% of women do not attend postpartum care visits, with lower attendance rates among patient populations with limited healthcare access (19, 20).

Marijuana-exposed participants exhibited characteristics reflecting those reported in the existing literature. We found that women with lower education levels used marijuana in pregnancy at a higher frequency than those with higher educational backgrounds (21). Notably, 91% (n=21) of marijuana-exposed participants identified as non-Hispanic white. This demographic trend may have been influenced by the study’s reliance on patients self-reporting perinatal marijuana use and the subsequent assessment of patient interest by obstetric care clinicians. Available studies show that pregnant women of Black and Hispanic ethnicity, lower median neighborhood incomes, and older age are less likely to self-report (22, 23). This discrepancy may arise due to effects on social desirability, discrimination, and fear of legal/custodial consequences. This may suggest that obstetric care clinicians should take a proactive approach to provide further support and resources on marijuana use risks. Further research could examine socio-demographic differences in self-reports and the downstream effects that may arise from a lack of counseling on marijuana use and cessation during pregnancy.

Overall, we found that marijuana-exposed participants were interested in and agreeable to engaging in longitudinal research in the antepartum and postpartum periods. Participants demonstrated willingness to provide biospecimens and undergo imaging once they understood the purpose of the study and developed a rapport with the research staff. Limitations of this study include the small sample population relative to the large healthcare setting, although a sample size of approximately 20 was deemed sufficient for feasibility data. Future research could examine implementing staggered increased incentivization and home visits to improve retention and promote continued study participation. Additionally, reliance on hospital staff for biospecimen collection resulted in missed samples, underscoring the need for improved staffing, coordination, and follow-up. The work presented in this report should be used to improve recruitment/retention strategies in future studies to decrease participant attrition rates. Identifying trends in research participation rates of this subset of patients provides a key prerequisite to generating evidence-based guidelines for clinicians.

## Data Availability

The original contributions presented in the study are included in the article/supplementary material. Further inquiries can be directed to the corresponding author.
